# Further supporting evidence for *REEP1* phenotypic and allelic heterogeneity

**DOI:** 10.1212/NXG.0000000000000379

**Published:** 2019-11-15

**Authors:** Reza Maroofian, Mahdiyeh Behnam, Rauan Kaiyrzhanov, Vincenzo Salpietro, Mansour Salehi, Henry Houlden

**Affiliations:** From the Department of Neuromuscular Disorders Institute of Neurology (R.M., R.K., V.S., H.H.), University College London, Queen Square; and Medical Genetics Laboratory of Genome (M.B., M.S.), Isfahan, Iran.

Heterozygous mutations in *REEP1* (MIM #609139) encoding the receptor expression-enhancing protein 1 (REEP1) are a well-recognized and relatively frequent cause of autosomal dominant hereditary spastic paraplegia (HSP), SPG31.^1^ REEP1 localizes in the mitochondria and endoplasmic reticulum (ER) and facilitates ER-mitochondria interactions.^2^ In addition to the HSP phenotype, REEP1 has been associated with an autosomal dominant spinal type of Charcot-Marie-Tooth disease in 2 families.^3^ More recently, a patient with homozygous REEP1 mutation with a much more severe phenotype akin to spinal muscular atrophy with respiratory distress type 1 (SMARD1) was reported.^4^ In this report, we present a patient with a homozygous mutation in *REEP1* manifesting a severe congenital distal spinal muscular atrophy (SMA) with diaphragmatic paralysis, expanding the phenotype from mild autosomal dominant HSP through to severe recessive distal SMA pattern.

## Clinical report

The proband is a 14-year-old girl who is the product of a normal full-term pregnancy. Her parents are healthy first-cousin Iranians in their mid-thirties. At birth, she presented with proximal and distal muscle weakness in all the limbs with flexion contractures at the knees and elbows, foot drop, and progressive wrist drop (figure1, B–F, Video 1). She could crawl, hold her head erect at normal age, and sit at the age of 5 months. Afterward, there has been a marked regression in neurologic development, resulting in permanent inability to stand up and/or walk and in severe recurrent contractions in hands and feet despite surgery being started from the age of 2.5 years. She developed progressive muscular atrophy starting from the lower limbs and progressing to the upper limbs. At the age of 3 years, she was diagnosed with diaphragmatic palsy, resulting in fever-associated breathing difficulties. She was also diagnosed with esophageal sphincter palsy, which was corrected by surgery. Her cognition and speech are preserved, and she does not have visual or hearing impairment.

**Figure:**
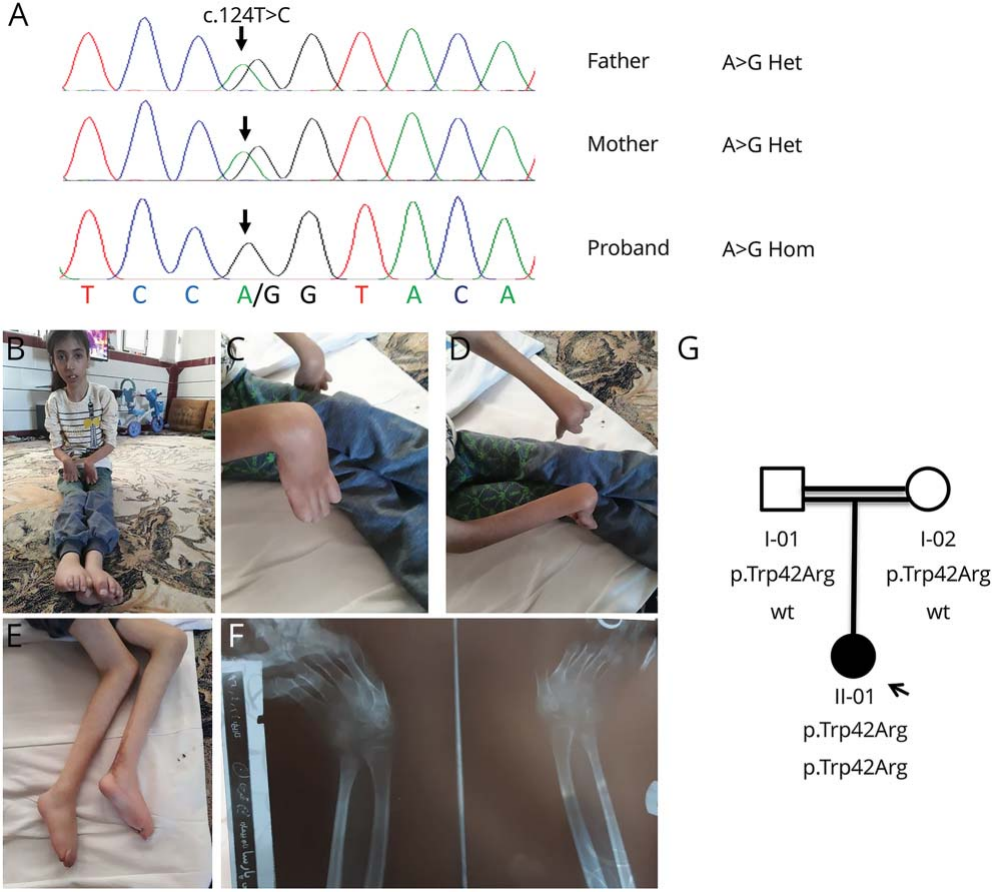
Clinical images and molecular genetic findings of the case (A) DNA chromatograms. The top row is a sequence from the father, the middle row is from the mother, and the bottom row is from the proband. The blue arrow points at A > G substitution, which is heterozygous in the parents and homozygous in the proband. (B–E) Distal and proximal arthrogryposis. Wasted limb muscles. (F) X-ray of the arms. Deformity of the middle phalanges and lateroflexion of the hand. (G) Family tree.

10.1212/000379_Video_1 Video 1 The reported patient is sits, and this is followed by an attempt to crawl, and then, she rolled over. Proximal and distal arthrogryposis, flexed trunk and head, and mild facial dystonia are present. Mobility is severely impaired. Download Supplementary Video 1 via http://dx.doi.org/10.1212/000379_Video_1

On neurologic examination, she had marked plantar flexion contractures in the ankles and flexion contractions in the wrists, brisk deep tendon reflexes in the knees, absent Achilles reflex, no Babinski sign, and normal reflexes in the upper limbs. There was low muscle tone and wasting in the limbs. Neurophysiology studies showed signs of severe sensory and moderate motor axonal degeneration, fibrillations, and marked reduction in the motor unit interference pattern with an increase in amplitude and duration of motor unit action potentials in the right and left tibialis anterior muscles. The latter change was also observed to a lesser degree in the right biceps and left deltoid. Sural nerve biopsy was compatible with chronic demyelinating neuropathy with secondary focal axonal and nerve fibers loss. Brain MRI was unremarkable. Dosage and Sanger sequencing of the *SMN1* gene was performed and resulted negative. The clinical diagnosis of distal SMA with diaphragmatic paralysis was made. Single-nucleotide polymorphism array genotyping in combination with exome sequencing revealed a homozygous pathogenic missense variant in *REEP1* (NM_022912: c.124T > C; p.Trp42Arg) within a 59 Mb homozygosity region (figure1A).

## Discussion

The p.Trp42Arg *REEP1* variant has been previously reported as pathogenic in autosomal dominant hereditary spastic paraplegia.^5^ The mutation leads to haploinsufficiency and interferes with the integrity of the N-terminus of REEP1 and impairs normal ER targeting and shaping.^6^ It has been speculated in a *Drosophila* model that *REEP1*-associated disorders could be ER stress diseases expressing variable phenotypes depending on the type of impaired function in the ER.^4^ Furthermore, REEP1 could have a direct role in the control of mitochondrial network morphology through interaction with the crucial protein involved in mitochondrial fission, named DRP1. It has been shown that important alterations take place in the mitochondrial morphology in the primary fibroblasts of patients with REEP1 HSP compared with control cells.^7^

To date, only 1 homozygous mutation has been reported in *REEP1*; this affects a splice donor site (p.Phe62Lysfs23*) and was found in a Lebanese patient presenting with a SMARD1-like phenotype which included distal arthrogryposis, congenital axonal neuropathy, hyperreflexia, and respiratory distress, but without any cognitive or language impairment.^4^ Although there was a significant phenotypic overlap between this reported individual and our patient, arthrogryposis involving proximal and distal joints was more pronounced in our patient, whereas diaphragmatic palsy seems to be more severe in the homozygous p.Phe62Lysfs23* carrier. In addition, our case adds data on peculiar electromyographic changes that were not reported in the former report. Of interest, parents were carriers of the variant in the heterozygous state, already associated with complex autosomal dominant hereditary spastic paraplegia, and neither gait problems nor family history of HSP was reported by them. REEP1 heterozygous mutations express incomplete penetrance predominantly manifesting before the age of 20 years or after the age of 30 years.^8^ Unfortunately, the parents did not have neurologic and electrophysiologic examinations to exclude the subclinical or mild forms of HSP. The incomplete penetrance of the p.Trp42Arg variant in the heterozygous state could be explained by the effects of modifying genes and stochastic processes during development.^4^

The identification of 2 different biallelic *REEP1* mutations in patients with SMARD1-like phenotypes and respiratory Distress expand the phenotypic spectrum associated to this gene and demonstrates the loss-of-function dosage effect and implications of the same allele in distinct neurologic phenotypes.
